# Human Case of *Rickettsia felis* Infection, Taiwan

**DOI:** 10.3201/eid1412.080525

**Published:** 2008-12

**Authors:** Kun-Hsien Tsai, Hsiu-Ying Lu, Jih-Jin Tsai, Sheng-Kai Yu, Jyh-Hsiung Huang, Pei-Yun Shu

**Affiliations:** Centers for Disease Control, Taipei, Taiwan, Republic of China (K.-H. Tsai, H.-Y. Lu, S.-K. Yu, J.-H. Huang, P.-Y. Shu); Kaohsiung Medical University, Kaohsiung, Taiwan, Republic of China (J.-J. Tsai)

**Keywords:** Rickettsia felis, human case, Taiwan, flea-borne spotted fever, letter

**To the Editor:** *Rickettsia felis*, the etiologic agent of flea-borne spotted fever, is carried by fleas worldwide (*1*). In the past decade, several human cases of *R. felis* infection have been reported (*1*–*3*). Clinical symptoms and biological data for *R. felis* infections are similar to those for murine typhus and other rickettsial diseases, which makes clinical diagnosis difficult (*2*). Patients with *R. felis* infections may have common clinical manifestations, such as fever, headache, myalgia, macular rash, and elevated levels of liver enzymes (*4*,*5*).

Reportable rickettsioses in Taiwan include scrub typhus, epidemic typhus, and murine typhus. Although there are no known human cases of infections caused by spotted fever group (SFG) rickettsiae in Taiwan, novel strains of SFG rickettsiae have been isolated as recently described (*6*,*7*). In addition, evidence for *R. felis* infections in cat and cat flea populations has been identified by using immunofluorescence assay (IFA), PCR, and organism isolation (K.-H.Tsai et al., unpub. data). We report an indigenous human case of *R. felis* infection in Taiwan.

In January 2005, a 27-year-old woman living in Fongshan City, Kaohsiung County, in southern Taiwan was admitted to Kaohsiung Medical University Hospital with a 4-day history of intermittent fever (37.8°C–38.0°C), chills, headache, and fatigue. Associated symptoms were frequent micturition and a burning sensation upon voiding. The patient was admitted with a possible urinary tract infection; urinalysis showed pyuria (leukocyte count 25–50/high-power field), compatible with the clinical diagnosis. During the 6-day hospital stay, the patient received daily intravenous first-generation cephalosporin (cefazolin); gentamicin was given only on the first 3 days. She was discharged with a prescription for oral antimicrobial drugs (cephradine 500 mg every 6 h) to be taken for 7 days. Micturition-associated symptoms subsided after treatment.

The patient also had headaches and glove-and-stocking numbness in both hands because of fever, but denied any associated rash and arthralgia. Although the patient did not recall any arthropod bites, she had noticed some stray dogs and cats nearby and rodents in the neighborhood surrounding her house. Because of acute polyneuropathy-like symptoms and exposure history, we prescribed oral doxycycline (100 mg every 12 hours) for 5 days as empirical therapy on the second day at the hospital, suspecting a zoonosis such as rickettsioses, Q fever, or leptospirosis. Headache and glove-and-stocking numbness subsided. Her blood sugar level and thyroid function were within normal limits. Chest radiograph, liver function, renal function, and levels of electrolytes were all normal.

Whole blood counts were normal, although differential counts demonstrated a left shift (polymorphonuclear leukocytes 80.7%, reference range 37%–75%). C-reactive protein level was 66.7 μg/mL, (reference <5 μg/mL) upon admission. Blood culture and urine culture were negative for bacteria.

Additionally, painful vesicles on the external genitalia appeared on the fourth day postadmission, and valacyclovir was administered for 5 days because of suspected infection with herpes simplex virus. The lesion subsided after valacyclovir treatment and the patient was discharged in good condition.

Patient whole blood specimens were collected on days 4 and 16 after the onset of fever and sent to the Taiwan Centers for Disease Control for laboratory diagnosis of rickettsial infection. For molecular diagnosis, DNA from the acute-phase blood sample (day 4) was analyzed by using the SYBR green-based real-time PCR specific for 17-kDa antigen, 60-kDa heat-shock protein (*groEL*) gene, and outer membrane protein B (*ompB*) gene for typhus group and SFG rickettsiae and primers listed in the Table. Nucleotide sequences of real-time PCR products demonstrated 100% identity with 17-kDa antigen, *groEL*, and *ompB* genes of *R*. *felis* URRWXCal2. Real-time PCR results were negative for *Orientia tsutsugamushi* and *Coxiella burnetii* (*8*).

**Table:** Primer sequences for the SYBR-based real-time PCR assay*

Primer	Genomic region	Sequence (5′ → 3′)	Amplicon size, bp
RR-F8	17-kDa (*Rickettsia* spp.)	GGC GGY GCA TTA CTT GGT TCT CAA TTC GC	304
RR-R12		GTT TTC CSC CTA TTA CAA CTG TTT GAG T	
RR-F1	*groEL* gene (*Rickettsia* spp.)	AAA ATG GTT GCT GAG CTT GAA AAT CCT TT	191
RR-R2		ACT TTC AAA CCA CCA CGT AAT CTA TTG AC	
RR-F22	*ompB* gene (*Rickettsia* spp.)	ATG GTR TAT GGG CWA AAC CTT TCT ATA	330
RR-R25		TAG MMT CGA AGA AGT AAC GCT GAC TTT	
RST-14F	56-kDa gene (*Orientia tsutsugamushi*)	CCA TTT GGT GGT ACA TTA GCT GCA GGT	233
RST-6R		TCA CGA TCA GCT ATA CTT ATA GGC A	
OMP3	*com-1* (*Coxiella burnetii*)	GAA GCG CAA CAA GAA GAA CAC	438
OMP4		TTG GAA GTT ATC ACG CAG TTG	

*groEL, 60-kDa heat-shock protein; *ompB*, outer membrane protein B; *com-1*, 27-kDa outer membrane protein.

For serologic diagnosis, serum samples were tested for rickettsial-specific antibodies by IFA using whole cell antigens of *R. felis* isolated from the cat flea. The patient’s serum (days 4 and 16) had immunoglobulin (Ig) G, IgA, and IgM titers of 160 and 2,560, respectively. The serum sample collected from *R. felis*–infected cat served as the positive control. Test results were negative for *R. typhi*, *R. conorii*, *R. rickettsii*, *R. japonica*, *O. tsutsugamushi*, and *C. burnetii*.

Absence of rash, eschar, and unawareness of arthropod bite may be easily overlooked in some patients with rickettsial infections. In this case, suspicion of rickettsial infection was based on exposure history and acute polyneuropathy, which responded quickly to doxycycline treatment. There are limited reports of rickettsioses with polyneuropathy, and none for cases of *R. felis* infection (*9*,*10*). It was hard to tell whether the urinary tract and herpes simplex virus infections were associated with an *R. felis* infection, but it is quite rare for 3 different infections to occur in a patient at the same time as isolated entities. The finding of a human case of infection and the existence of *R. felis* in cat fleas highlights the need for further studies on flea-borne rickettsioses in Taiwan.
